# Cyclic-di-GMP signalling mutants drive ecological succession and self-generated diversity in experimentally evolved biofilms of Pseudomonas aeruginosa

**DOI:** 10.1099/mic.0.001605

**Published:** 2025-09-15

**Authors:** Gregory J. Wickham, Chuanzhen Zhang, Ryan Sweet, Maria Solsona-Gaya, Mark A. Webber

**Affiliations:** 1 Quadram Institute Bioscience, Norwich Research Park, Norwich, NR4 7UQ, UK; 2 Earlham Institute, Norwich Research Park, Norwich, NR4 7UZ, UK; 3 Centre for Microbial Interactions, Norwich Research Park, Norwich, NR4 7UG, UK; 4 College of Veterinary Medicine, South China Agricultural University, Guangzhou, Guangdong, 510642, PR China; 5 Shanghai Public Health Clinical Centre, Fudan University, Shanghai, 201058, PR China; 6 Division of Blood and Marrow Transplantation and Cellular Therapy, Stanford University School of Medicine, Stanford, CA94304, USA; 7 Norwich Medical School, University of East Anglia, Norwich, NR4 7TJ, UK

**Keywords:** diguanylate cyclase, emergent property, experimental evolution, hyperbiofilm, niche adaptation, phosphodiesterase

## Abstract

Biofilms represent a discrete form of microbial life which are physiologically distinct from free-living planktonic cells. The altered phenotypic manifestations of the biofilm may also elicit lifestyle-dependent adaptive responses to selective pressures. In this work, an experimental evolution model was used to study the adaptation to a biofilm lifestyle in *Pseudomonas aeruginosa* PA14. The serial passage of biofilms selected for biofilm hyperproduction in a stepwise fashion characterized by increased biomass production and phenotypic diversification was not associated with reduced susceptibility to antibiotics. Adaptation to a biofilm lifestyle selected for mutations causes constitutive increases of intracellular c-di-GMP concentrations via mutations in the phosphodiesterase *dipA*, the *yfiBNR* signalling complex and the bifunctional diguanylate cyclase/phosphodiesterase *morA*. Furthermore, selection for biofilm hyperproduction also gave rise to self-generated diversity by eliciting morphotypic diversification into complex community structures. Individual morphotypes were not associated with specific mutations and lineages dynamically switched between morphotypes despite possessing conserved mechanisms of biofilm hyperproduction. This work provides insights into the evolutionary importance of self-generated diversity to the biofilm and reveals the genetic control and phenotypic dynamics which contribute to the characteristically rugged fitness landscape associated with a sessile lifestyle.

## Data Availability

Genome sequences were either publicly available and downloaded from the National Center for Biotechnology Information (NCBI, https://www.ncbi.nlm.nih.gov) or obtained from strains sequenced in this study. Raw reads generated from this work can be found on the NCBI under individual SRA accession numbers SRR32576768–SRR32576815 available from BioProject PRJNA1232141.

## Introduction

Bacterial biofilms are complex microbial structures comprised of cells confined within a secreted matrix of protein, polysaccharides and extracellular DNA [1]. Biofilms that form on inert surfaces possess a life cycle characterized by the formation of aggregate microcolonies which mature into complex biofilms and, in turn, disperse to colonize new environments. The transition from a free-living planktonic state to a sessile biofilm confers broad phenotypic changes for both the individual cells and the wider community known as ‘emergent properties’ [2]. The composite architecture, population heterogeneity and social interactions given rise by the biofilm lifestyle contribute to their widely described environmental persistence which presents significant challenges in both clinical and industrial environments [3]. It is estimated that up to 70% of bacterial infections possess a biofilm component somewhere in the chain of infection, from contamination of fomites to direct colonization of indwelling medical devices [4,5]. Over half of healthcare-acquired infections can be attributed to contamination of medical devices, which are highly amenable to biofilm-associated infections as they grant a route of entry to sterile sites. Furthermore, they also provide a non-shedding surface to colonize which abrogates the body’s colonization resistance through cellular turnover [6,7]. Biofilm-associated infections are consequently more challenging to resolve with antimicrobial therapy alone and increase the risk of disseminated disease.

Biofilm-associated infections are often characterized by long-term chronicity which facilitates evolutionary change over a single infective period [8]. Strains isolated from chronic biofilm infections often demonstrate mucoidy, sessility and phenotypic heterogeneity, suggesting that the biofilm lifestyle may offer a selective advantage for subclinical persistence. Notably, chronic infections of *Pseudomonas aeruginosa* in cystic fibrosis lung are associated with extensive within-host evolution [9]. This is best characterized by phenotypic diversification of cultured isolates which includes mucoid conversion, hypermutators and small colony variants (SCVs), driven by a plethora of mutations in biofilm-associated genes such as *wspF* and *yfiBNR* [10]. The clinical importance of this phenomenon is highlighted by reports of reduced susceptibility to antibiotics, impaired lung function and protracted disease durations associated with long-term carriage of *P. aeruginosa* in the cystic fibrosis lung [11]. Furthermore, an increased competence in biofilm formation is broadly linked with greater persistence of *Listeria monocytogenes* and salmonellae in food processing environments and *P. aeruginosa* and *Legionella pneumophila* in hospital plumbing systems [12,14]. Indeed, up to half of *P. aeruginosa* infections in intensive care units can be attributed to biofilm-contaminated water systems which transmit pathogens from cryptic sources [15,16]. However, whether environmental conditions can select for biofilm-adapted lifestyles, or if strains become environmentally persistent having already acquired increased biofilm competence beforehand, remains unclear.

In *P. aeruginosa,* biofilm formation is regulated through a complex network of signalling cascades which control the transition from a motile planktonic state to a sessile lifestyle in distinct transcriptional stages [17]. One of the most important regulatory components controlling this in *P. aeruginosa* is the bacterial second messenger cyclic diguanylate (c-di-GMP). The intracellular c-di-GMP reservoir is controlled by a sophisticated network of diguanylate cyclases and phosphodiesterases which act to enrich or deplete concentrations of c-di-GMP, respectively [18]. Elevated c-di-GMP concentrations activate signalling cascades such as Gac/Rsm, which supports biofilm formation by upregulating the secretion of exopolysaccharides and repressing flagellar motility [19,20]. When environmental conditions change favouring return to a planktonic lifestyle, the net phosphodiesterase activity increases, leading to reduced extracellular matrix production and increased motility, facilitating the release of cells from the biofilm [21]. This dynamic enables *P. aeruginosa* to transition between biofilm-associated and planktonic lifestyles in response to environmental changes. However, mutations that result in constitutive increases in intracellular c-di-GMP levels can confer sustained increases in biofilm formation to improve fitness during physicochemical stress such as fluid shear, temperature fluctuations and antimicrobial insults.

Beyond the immediate fitness benefits, commitment to a biofilm lifestyle also introduces fundamental changes in fitness dynamics which may elicit divergent selective outcomes, whereby traits under selection which possess lifestyle-dependent fitness effects alter evolutionary trajectories. For instance, when members of a population are functionally independent, such as during planktonic growth, it is often more advantageous to selectively exclude competitors than to cooperate [22]. However, when obligately localized within a community that shares space, nutrients and adverse conditions, it can be more beneficial to cooperate to achieve compound increases in fitness [23]. Specifically, adaptation to a biofilm lifestyle carries two main evolutionary hallmarks to achieve higher-order fitness gains, ecological diversification and development of social traits, which have been widely observed *in vitro* [24,27]. Consequently, the biofilm lifestyle can offer selective advantages by revealing alternative adaptive walks on the fitness landscape unavailable to planktonic individuals, which may have profound implications in defining evolutionary trajectories. However, how these traits are generated across different environments and their downstream implications on other phenotypes are less clear.

*P. aeruginosa* readily forms biofilms in laboratory settings and is therefore a widely used model organism to investigate biofilm biology. In this work, we leveraged an experimental evolution model originally described by Poltak and Cooper [28] to adapt lineages of * P. aeruginosa* grown as biofilms on bead substrata of glass, polyvinyl chloride (PVC) and type-319 stainless steel through serial passage. Phenotypic changes in biofilm formation, population heterogeneity, fitness, antimicrobial susceptibility and stress tolerance of the evolved lineages from the ancestor were assayed in adapted lineages over the course of the transfer series. Further, genome sequencing of the experimentally evolved lineages was performed at each assayed timepoint, and the evolutionary trajectories associated with biofilm hyperproduction on each substratum material were characterized. This work demonstrates the importance of cyclic-di-GMP signalling pathways in the selection of biofilm hyperproduction and reveals the mechanisms by which successive increases in biofilm formation are achieved. Furthermore, it also highlights the fundamentality of self-generated diversity to the biofilm lifestyle, which switched dynamically between morphotypes despite conserved mechanisms of biofilm hyperproduction.

## Methods

### Experimental evolution

Biofilms of *P. aeruginosa* PA14 were cultured on 5 mm PVC (Xinlin Industrial Company Ltd, Ningbo, China), glass (Sigma-Aldrich Corporation, Gillingham, UK) or type-316 stainless steel (Simply Bearings Ltd, Leigh, UK) beads in untreated 24-well cell culture plates (Eppendorf UK Ltd, Stevenage, UK) containing 1 ml lysogeny broth (Oxoid, Basingstoke, UK) shaking at 60 r.p.m. for 48 h at 30 °C. Each well contained three beads: one bead was stored in 1 ml 20% v/v glycerol in a 2 ml 96-well deep-well plate [Starlab (UK) Ltd., Milton Keynes, UK], one bead was used to propagate to the next transfer and the third bead was a spare. The beads were moved using a sterile 10 µl inoculation loop (Fisher Scientific, Loughborough, UK) and, at each timepoint, were washed in 1 ml PBS with gentle agitation before being transferred into fresh broth containing three more sterile beads of a different colour ready to seed for the next transfer. In this way, biofilms were serially passaged by transferring seeded beads to fresh wells containing three sterile beads allowing biofilms to passively disseminate to a fresh substrate at each timepoint. Four lineages of biofilms were passaged for 30 passages on each of the test substrata in addition to four lineages of planktonic controls propagated in wells without beads via a 1 : 100 dilution into fresh media. At the end of the passage series, biofilms were recovered from the glycerol bead archives by sealing plates with 96-well sealing mats (Thermo Fisher Scientific, Basingstoke, UK) and vortexing at high speed for ~1 min. Each of the four lineages adapted to each selective substrate and planktonic growth at transfers 10, 20 and 30 was resuscitated for phenotyping and genome sequencing. This resulted in 4 isolates per timepoint, per selective condition, and 48 isolates in total, examined for phenotypic and mutational changes over the course of the evolution experiment.

### Biofilm productivity assay

Overnight cultures were diluted 1 : 100 into 1 ml of selected microbiological culture medium in an untreated 24-well cell culture plate containing beads of a chosen substrate and incubated at 25 °C, shaking at 60 r.p.m. for 48 h. Beads were then washed twice in 1 ml PBS and transferred to a 1.5 ml microcentrifuge tube (Fisher Scientific) containing 1 ml PBS. Cells were harvested via agitation at 2,000 r.p.m. for 2 min in an Eppendorf ThermoMixer (Eppendorf UK Ltd). The harvested cells were then diluted 1 : 10 into the first row of a microtitre tray (Greiner Bio-One, Kremsmünster, Austria) containing 180 µl PBS and serially diluted down the columns of the tray. After dilution, 5 µl of each well was spotted onto a square lysogeny broth (LB) agar plate (R and L Slaughter Ltd, Basildon, UK) which was incubated for 24 h at 37 °C. The number of c.f.u. was then calculated per unit area.

### Crystal violet biomass assay

Overnight cultures were diluted 1 : 10,000 by diluting 1 : 100 twice in microtitre trays containing 180 µl of LB. The inoculated plates were sealed with a gas-permeable membrane (4titude Ltd, Wotton, UK) and incubated for 48 h at 30 °C. After incubation, the culture was discarded, and the wells were washed several times with tap water. Into each well, 200 µl of 0.1% crystal violet solution (Sigma-Aldrich Corporation) was added and incubated at room temperature for 15 min. The crystal violet was then discarded, and the wells were again washed several times with tap water. Finally, 70% ethanol (Sigma-Aldrich Corporation) was added to each well, and the OD as measured by absorbance at 595 nm (OD_595_) was read in a FLUOstar Omega plate reader (BMG Labtech, Aylesbury, UK).

### Congo red-Coomassie blue colony morphology assay

Biofilm morphology agar plates containing 1% w/v bacteriological agar, 1% w/v tryptone, 40 µg ml^−1^ Congo red (Sigma-Aldrich Corporation) and 20 µg ml^−1^ Coomassie Brilliant Blue-R (Sigma-Aldrich Corporation) were poured into 50 mm Petri dishes (R and L Slaughter Ltd). Overnight cultures were diluted to an OD_595_ of 0.5 (±0.05) in PBS using a Jenway 7200 spectrophotometer (Cole-Parmer, Stone, UK), and 10 µl was spotted onto biofilm morphology agar plates. The plates were incubated at 20 °C for 10 days and then imaged using a Canon PowerShot SX210 digital camera [Canon (UK), Reigate, UK]. Morphometric properties including colony area, agar invasion area and colony rugosity were determined using Fiji v2.1.0 [29].

### MIC determination by microbroth dilution

Minimum inhibitory concentration (MIC) determination by microbroth dilution was performed following European Committee for Antimicrobial Susceptibility Testing (EUCAST) recommendations [30]. The second to last columns of a microtitre tray were filled with 100 µl Mueller–Hinton broth. In the first and second columns of the microtitre tray, 100 µl of the selected antimicrobial at 128-times MIC in Mueller–Hinton broth was added. The antibiotic was then double diluted across column 2 to column 11. Column 12 was left without antibiotic as a control. Overnight cultures were diluted 1 : 100 twice in Mueller–Hinton broth, and 100 µl was added to each well producing a dilution series from 64-times MIC to 0.06-times MIC. The plate was sealed with a gas-permeable membrane and incubated at 37 °C for 24 h. The lowest concentration in which no growth was observed was designated as the MIC.

### Minimum biofilm eradication concentration determination

A modified broth microdilution assay was used to determine the minimum biofilm eradication concentration (MBEC). Overnight cultures were diluted 1 : 100 twice in microtitre trays containing 180 µl of LB. A skirted 96-well PCR plate (Starlab, Milton Keynes, UK) was then allowed to sit within the wells of the microtitre tray. The plate was incubated for 48 h at 30 °C, allowing a biofilm to form on the outside of the wells of the PCR plate. After incubation, the microtitre tray was discarded, and the outside surface of the wells of the PCR plate was washed in 200 µl of PBS in a microtitre tray. The PCR plate was then allowed to rest within another microtitre tray containing antimicrobials in the same format as the MIC assay described previously, which was incubated at 37 °C for 24 h. The MIC plate was then discarded, and the PCR plate was placed in a fresh microtitre tray containing 200 µl of LB which was incubated for 24 h at 37 °C. The corresponding concentration from the first well which the biofilm could not reseed was determined to be the MBEC.

### Stress tolerance assays

Strains were grown overnight, diluted 1 : 1,000 into LB containing 9% w/v sodium chloride or in LB adjusted to either pH 4 or pH 12. In a microtitre tray, 200 µl of the diluted stock was added and incubated at 30 °C for 48 h. The cultures were then serially diluted in PBS, and 5 µl of diluent was spotted onto a square LB agar plate and incubated at 37 °C for 24 h. The viability of each strain after stress challenge was determined by counting c.f.u.

### Fitness assay by growth kinetics

Overnight cultures were diluted 1 : 1,000 into a microtitre tray in 200 µl of LB. The plate was incubated for 20 h at 37 °C, and the OD_600_ was measured every 15 min using a FLUOstar Omega plate reader. The plate was homogenized by orbital shaking at 300 r.p.m. for 3 s before each read. The integral area under the curve (AUC) (OD_600_ hours) was calculated, and the exponential phase growth velocity was calculated.

### DNA extraction and quantification

Extraction of genomic DNA was performed with the Zymo Quick-DNA 96-Well Kit (Zymo Research, Irvine, CA, USA). Strains were grown overnight, and 2 ml of culture was transferred to a well of a deep-well plate. The deep-well plate was centrifuged for 15 min at 3,000 ***g*** to pellet cells, and the supernatant was discarded. The cells were resuspended in 400 µl BashingBead buffer and transferred to the tubes of a ZR BashingBead Lysis Rack. The tubes were vortexed at high speed for 10 min, and 250 µl of the BashingBead buffer was transferred to the wells of a 96-well block along with 750 µl of genomic lysis buffer. The 96-well block was sealed and vortexed at high speed for 5 min and centrifuged at 3,000 ***g*** for another 5 min. A silicon-A plate was mounted on a collection plate, and 500 µl of lysed cells was transferred into the wells of the silicon-A plate. The assembly was centrifuged at 3,000 ***g*** for 5 min, and the flow-through was discarded. This was repeated for the remaining 500 µl of lysed cells, followed by 200 µl of DNA pre-wash buffer and 500 µl of gDNA wash buffer. The collection plate was then replaced with an elution plate, and the DNA was eluted into it with 50 µl of elution buffer. The silicon-A plate was discarded, and the elution plate containing purified DNA was stored at −20 °C.

Extracted DNA was quantified using a Quant-iT dsDNA high sensitivity assay kit (Thermo Fisher Scientific). The Quant-iT working solution was made by diluting the Quant-iT reagent 1 : 200 into the Quant-iT buffer. A standard curve was generated by adding 10 µl of standard solution to 190 µl of working solution, and 2 µl of extracted DNA was added to 198 µl of working solution. Fluorescent excitation and emission wavelengths were measured at 500/530 nm. Fluorescent readings for extracted DNA were interpolated into the standard curve to yield DNA concentrations.

### Whole-genome sequencing

Whole-genome sequencing was performed using the Illumina NextSeq 500 platform (Illumina, Cambridge, UK). Extracted gDNA was diluted to 5 ng µl^−1^ in ultrapure deionized water, and sequencing libraries were prepared according to Illumina DNA Prep Reference Guide (Illumina, document #1000000025416 v10, 2020). A tagmentation master mix was created on ice containing 0.5 µl bead-linked transposomes, 0.5 µl tagmentation buffer 1 and 4 µl ultrapure water per sample, and 2 µl of gDNA was added to 5 µl of master mix. The gDNA was then tagmented at 55 °C for 15 min and dual indexed with 1 µl of i5 and i7 primers. In each well, 2 µl ultrapure water, 10 µl KAPA2G Fast ReadyMix (Roche, Welwyn Garden City, UK) and 7 µl of the tagmented gDNA were added. Barcoding was performed via PCR by initially denaturing at 95 °C for 1 min, followed by 14 cycles of denaturing at 95 °C for 10 s, annealing at 55 °C for 20 s and extending at 72 °C for 3 min. The indexed sequencing libraries were then quantified as described previously and purified using AMPure XP solid phase reversible immobilization beads (Sigma-Aldrich Corporation). Libraries were normalized to 2 ng µl^−1^ and pooled in equal quantities and run on a High Sensitivity D1000 ScreenTape with an Agilent Tapestation 4200 to calculate the molar concentration of the final library pool at the 300–700 bp insert size region. The libraries were combined in equal volumes with 0.2 M sodium hydroxide and 200 mM Tris-hydrochloric acid and diluted to 20 pM in chilled hybridization buffer. The libraries were then further diluted to 1.8 pM in hybridization buffer, made up to a final volume of 1.3 ml with 1% PhiX Control v3 spike-in and were loaded into 300 cycle high-output NextSeq 500/550 kit (Illumina) for sequencing following Illumina’s recommended denaturation and loading protocol.

### Bioinformatics and statistical analysis

Demultiplexing of sequencing libraries and generation of separate fastq files were carried out on BaseSpace v6.16 (Illumina). Illumina adapters and low-quality reads were trimmed using Trimmomatic v0.38.0 [31] using default settings. Reads were assembled into contigs with Shovill v1.1.0 (https://github.com/tseemann/shovill) using SPAdes, and assembly quality was assessed using QUAST v5.0.2 [32]. Genomes were annotated using Prokka v1.14.5 [33]. SNP calling and core genome alignment were performed using paired reads with snippy v4.4.3 (https://github.com/tseemann/snippy) against a *P. aeruginosa* UCBPP-PA14 reference genome (GenBank accession GCF_000014625.1). Statistical analysis was performed in RStudio v2023.12.0+369 [34] using R 4.3.1. Data were visualized using ggplot2 v3.3.5 [35]. All statistical analysis and data visualization scripts are available at https://github.com/gwickh/biofilm_hyperproduction_paper.

## Results

### Biofilm hyperproducers form more biomass but do not become more productive or less fit in planktonic culture

**Fig. 1.**
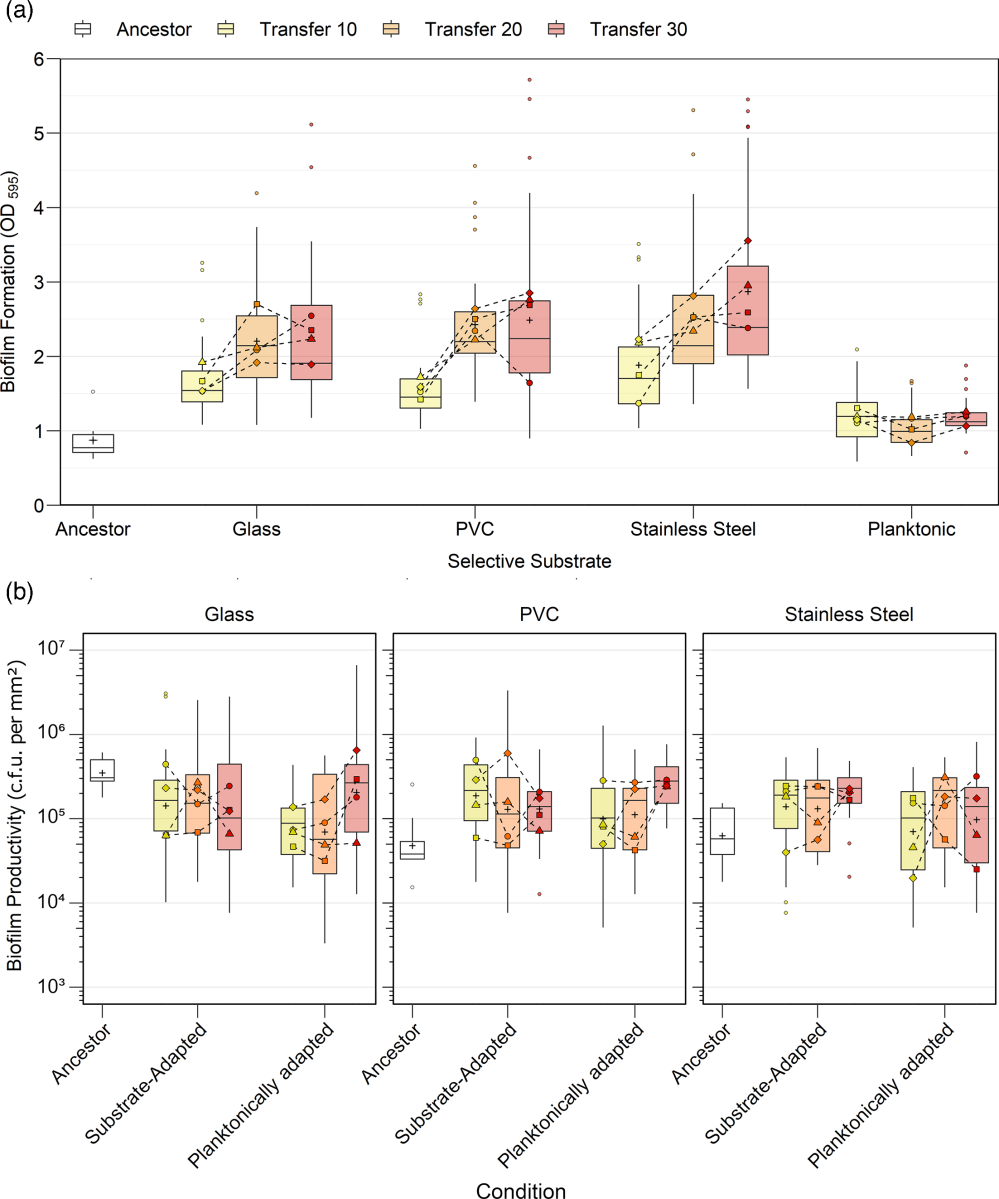
(a) Biofilm formation as measured via crystal violet staining of experimentally evolved lineages of *P. aeruginosa* adapted to growth planktonically or as a biofilm on glass, PVC or stainless steel substrates in LB for 30 transfers. Statistical differences between groups were determined via a two-way ANOVA at a 0.05 significance level. Significant main effects of substrate [*F*(4, 379)=45.89, *P*<0.0001] and number of transfers [*F*(2, 379)=25.32, *P*<0.0001] on biofilm formation were identified. Post hoc testing with Šidák correction identified significant increases in biofilm formation relative to the ancestor by transfer 20 in all biofilm-adapted lineages (glass: *P*=0.0005, PVC: *P*<0.0001, stainless steel: *P*<0.0001). Further increases were observed from transfer 20 to transfer 30 in all selective substrates except glass (glass: *P*=0.0578, PVC: *P*<0.0001, stainless steel: *P*<0.0001). No significant change was observed at any timepoint in the planktonically adapted lineages (transfer 10: *P*=0.9973, transfer 20: *P*>0.9999, transfer 30: *P*=0.9978). (b) Cellular productivity of experimentally evolved lineages grown on their selective substrate. A one-way ANOVA found no significant effect of selective substrate on productivity [*F*(2, 591)=1.821, *P*=0.1628]. Data are shown as (a) mean OD_595_ and (b) as mean c.f.u. per mm^2^, box limits show first and third quartiles, whiskers show ±1.5× interquartile range, large points show lineage mean and small points show outliers, *n*=16.

After 30 transfers, the biofilm-adapted lineages produced on average 2 to 3 times more biomass than the ancestral strain across all 3 selective substrates (Fig. 1a). Significant effects of both substrate and number of transfers on biofilm formation were detected (*P*<0.0001). Significant increases in biofilm formation relative to the ancestor were observed by transfer 20 in all biofilm-adapted lineages (glass: *P*=0.0005, PVC and stainless steel: *P*<0.0001). Further increases were observed by transfer 30 in lineages adapted to all selective substrates except glass (*P*<0.0001), indicating that instead of a single selective event, biofilm hyperproduction largely evolved in a stepwise manner. No significant change in biofilm formation relative to the ancestor was observed at any timepoint in the planktonically adapted lineages (*P*>0.999). Biofilm hyperproduction had little effect on cellular productivity, as measured as c.f.u. mm^2^ of substrate (Fig. 1b). Biofilms cultivated on beads could support ~10^5^ c.f.u. mm^2^, and despite increasing biofilm formation, a significant effect of the number of transfers on productivity was not detected (*P*=0.1628).

**Fig. 2.**
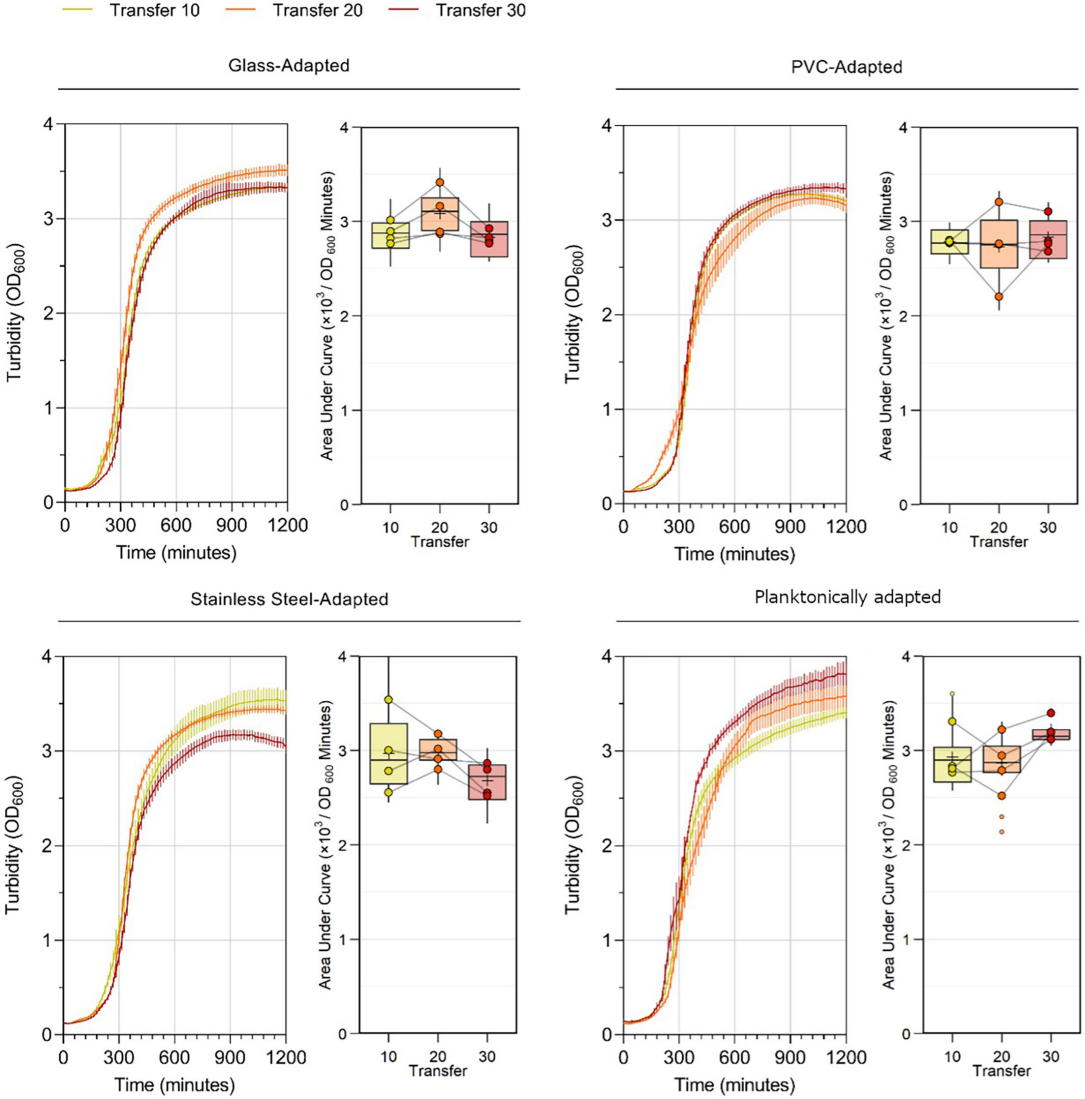
Growth kinetics of experimentally evolved lineages during planktonic growth. Statistical differences between groups were determined via a two-way ANOVA. A significant main effect of selective substrate on AUC [*F*(3, 180)=5.508, *P*=0.0010] and a significant interaction effect associated with substrate and number of transfers [*F*(6, 180)=5.895, *P*<0.001] were identified; however, no main effect of transfers [*F*(2, 180)=0.162, *P*=0.8510] was detected. Post hoc testing with Šidák correction identified that all biofilm-selective conditions were significantly less fit than the planktonically adapted lineages by transfer 30 (*P*<0.0001) driven by an increase in fitness in the planktonically adapted lineages between transfers 20 and 30 (*P*=0.0040). Adaptation to a biofilm lifestyle was not associated with a reduction in fitness from baseline (glass: *P*>0.9999, PVC: *P*>0.9999, stainless steel: *P*=0.0590). Curves show mean OD_600_±sd. Boxplot shows AUC, box limits show first and third quartiles, whiskers show ±1.5× interquartile range, large points show lineage mean and small points show outliers, *n*=16.

Gross fitness changes were quantified by measuring the AUC of growth kinetics in broth (Fig. 2). A significant effect of selective substrate (*P*=0.0010) and a significant interaction effect between substrate and number of transfers (*P*<0.0001) were detected. All biofilm substrates selected for lineages were significantly less fit than the planktonically adapted lineages by transfer 30 (*P*=0.0001), which was largely driven by an increase in fitness in the planktonically adapted lineages between transfers 20 and 30 (*P*=0.0040). Though fitness gains were constrained by adaptation to a biofilm lifestyle, it was not associated with a reduction in fitness (glass and PVC: *P*>0.9999, stainless steel: *P*=0.0590).

### Biofilm hyperproduction does not confer reduced susceptibility to antibiotics but does result in increased tolerance to salt stress and increased susceptibility to basic pH

Despite the adapted strains producing significantly more biofilm, this did not result in altered susceptibility to piperacillin, ceftazidime, meropenem, aztreonam, tobramycin or colistin according to the MIC or MBEC (Table S1, available in the online Supplementary Material). However, a significant increase in viability in media supplemented with 9% w/v salt was detected in biofilm-adapted lineages after 30 transfers (*P*<0.0001). The ancestor and the planktonically adapted lineages exhibited a non-significant 3- to 4-log_10_ reduction in viability (*P*=0.1020), whereas the biofilm-adapted lineages exhibited a significantly lower 2- to 3-log_10_ reduction across all selective substrates (*P*<0.0001, Fig. S1). No significant differences were detected between the biofilm-adapted groups themselves.

The evolved strains were also examined for altered tolerance to high and low pH conditions (Fig. S2). A significant difference in viability at pH 4 and 12 between selective substrates after 30 transfers was detected (*P*<0.0001), and a significant interaction effect between pH and selective substrate was also observed (*P*<0.0001). At pH 4, all selective conditions, including the planktonically adapted lineages, demonstrated significantly higher viability than the ancestor (*P*<0.0001), likely a reflection of adaptation to the culture conditions. Conversely, at pH 12, all selective conditions demonstrated significantly lower viability than the ancestor (*P*<0.0001). Despite this, the biofilm-adapted lineages remained significantly more susceptible to alkaline stress than the planktonically adapted lineages (*P*<0.0001) but not to acid stress (glass: *P*=0.8127, PVC: *P*=0.9456, stainless steel: *P*=0.7509).

### Biofilm hyperproduction is associated with dynamic switching between complex colony morphotypes which possess increased rugosity and decreased agar invasion

**Fig. 3.**
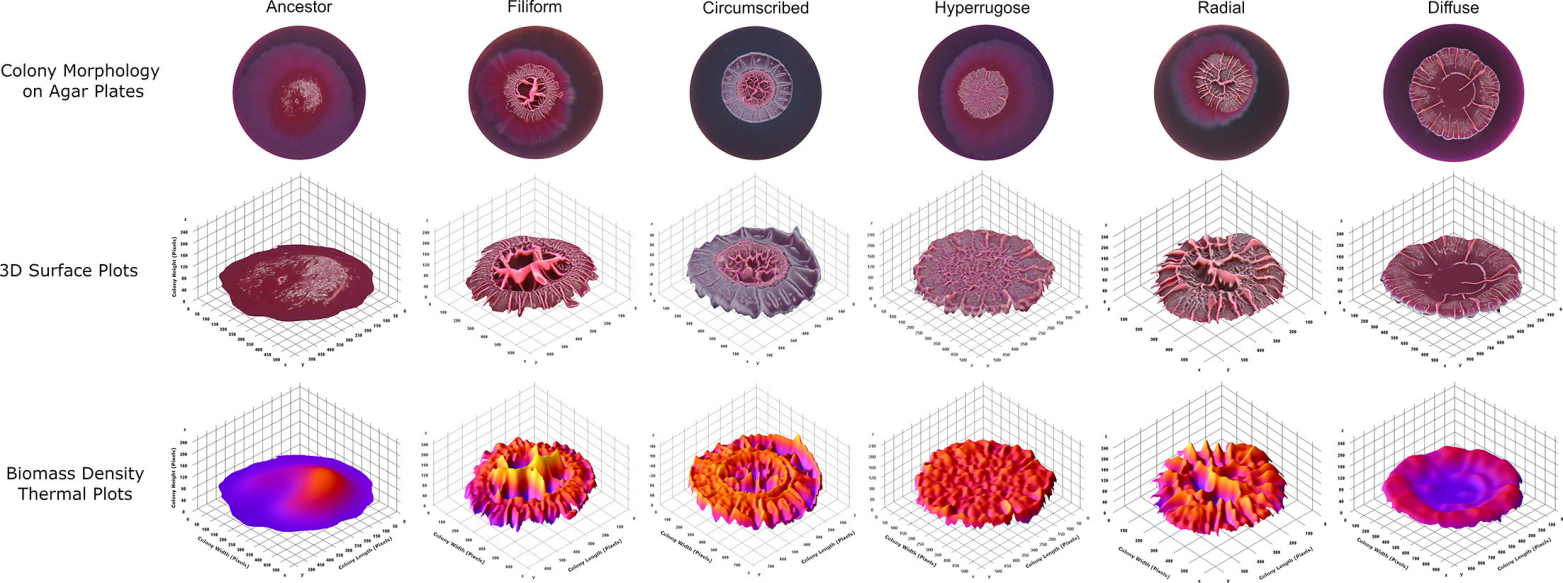
Colony morphotypes identified from experimentally evolved lineages after a 10-day incubation at 20 °C on bacteriological agar supplemented with 1% w/v tryptone, 20 µg ml^−1^ Congo red and 40 µg ml^−1^ Coomassie brilliant blue. Data are shown as representative colony dimensions in pixels, *n*=4.

The colony morphologies of the biofilm hyperproducers were investigated by plating populations on bacteriological agar supplemented with tryptone, Coomassie brilliant blue and Congo red (Fig. 3). After 10 days of incubation at room temperature, the ancestor formed a planar colony which was surrounded by a large halo of bacterial growth invading across the agar. In the biofilm-adapted lineages, a series of morphotypic variants were characterized which possessed distinct, complex colony architectures qualitatively distinguished based on Congo red dye uptake, colony size, agar invasion and rugosity patterns. The morphotypes assigned as filiform and circumscribed were characterized by large central folds of rugosity with a clearly delineated colony wall bordering the colony. Whereas the colony wall of the filiform morphotype was composed of many filamentous folds, the wall of the circumscribed morphotype was an uninterrupted assemblage of biomass with high Congo red uptake. Unlike the filiform and circumscribed morphotypes, the hyperrugose, radial and diffuse morphotypes did not possess a defined border distinguishing an internal and enclosing colony wall. Instead, folds tended to radiate outwards to the colony perimeter. The hyperrugose morphotype was smaller in size and possessed many small folds evenly distributed across the colony, whereas the diffuse morphotype was larger and possessed few large folds across the radius of the colony. The radial morphotype appeared to be an intermediate state with moderate rugosity between the latter two morphotypes.

**Fig. 4.**
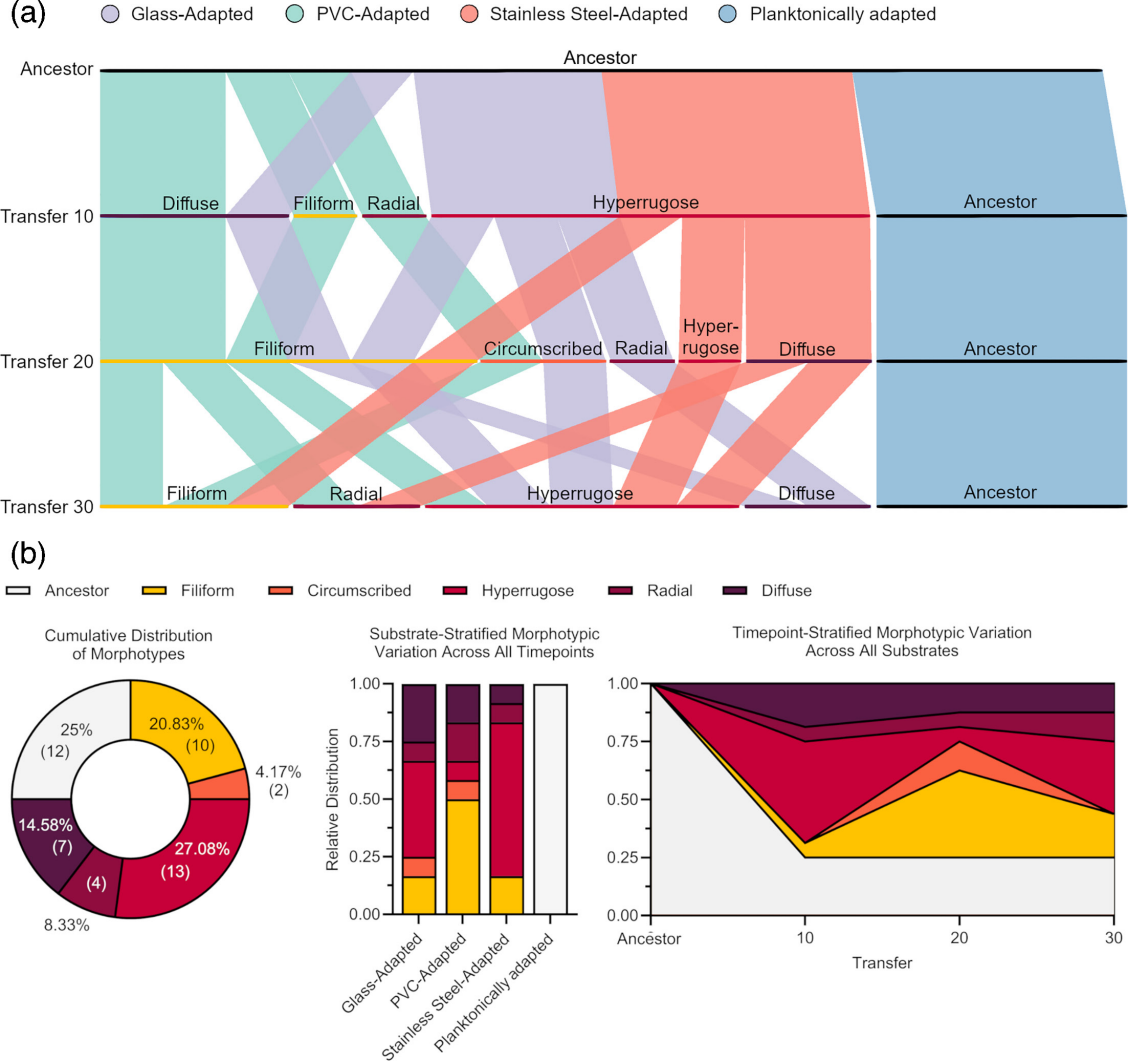
(a) Succession of colony morphotypes in experimentally evolved lineages. The morphotypic trajectory of each lineage was plotted using parallel sets v2.1. (b) Distribution of morphotypes across selective substrates and timepoints. Associations between colony morphotypes and selective substrate and timepoint were identified with a chi-square test for independence from 500,000 Monte Carlo simulations. Significant associations between both selective substrate and number of transfers on morphotype presentation were detected (substrate: *χ*^2^=241.56, *P*<0.0001, transfer: *χ*^2^=51.574, *P*<0.0001). Specific combinations driving these effects were determined using a *Z*-test for proportions on standardized residuals from the chi-square analysis controlling the false discovery rate using the Benjamini–Hochberg procedure. Hyperrugose colonies were significantly more likely in steel-adapted (residual=5.63, *P*<0.0001) and glass-adapted lineages (residual=2.63, *P*=0.0189) but were significantly underrepresented in PVC-adapted lineages (residual=−3.38, *P*=0.0020). Filiform and radial morphotypes were significantly overrepresented in PVC-adapted lineages (filiform: residual=5.75, *P*<0.0001, radial: residual=2.41, *P*=0.0293), and the diffuse morphotype was significantly associated with glass-adapted lineages (residual=2.3611, *P*=0.0312). The circumscribed morphotype, however, was not associated with any individual substrate (residual=1.6681, *P*=0.1271). At transfer 10, hyperrugose morphotypes were significantly overrepresented (residual=3.67, *P*=0.0011), while filiform morphotypes were significantly underrepresented (residual=−3.52, *P*=0.0016). At transfer 20, hyperrugose colonies were significantly underrepresented (residual=−4.59, *P*<0.0001) while both circumscribed and filiform morphotypes were strongly overrepresented (circumscribed: residual=4.09, *P*<0.0001, filiform: residual=4.02, *P*<0.0001). Data shown are as a proportion of observed morphotypes, *n*=4.

The planktonically adapted lineages retained an ancestral morphotype throughout the experiment; however, the biofilm-adapted lineages did not commit to a single morphotype, instead dynamically switching between morphotypes at different timepoints (Fig. 4). Overall, the hyperrugose and filiform morphotypes were the most commonly observed morphotypes in the biofilm-adapted lineages, with 13 and 10 unique observations, respectively. The diffuse, radial and circumscribed morphotypes were less common, with seven, four and two unique observations, respectively. A significant association between colony morphotype distribution and selective substrate was detected (*P*<0.0001). Specifically, hyperrugose colonies were significantly more likely in steel-adapted (*P*<0.0001) and glass-adapted lineages (*P*=0.0189) but were significantly underrepresented in PVC-adapted lineages (*P*=0.0020). Filiform and radial morphotypes were significantly overrepresented in PVC-adapted lineages (filiform: *P*<0.0001, radial: *P*=0.0293), and the diffuse morphotype was significantly more likely in the glass-adapted lineages (*P*=0.0312). The circumscribed morphotype, however, was not associated with any individual substrate (*P*=0.1271). Morphotype distribution was also significantly associated with timepoint (*P*<0.0001). At transfer 10, hyperrugose morphotypes were significantly overrepresented, while filiform morphotypes were significantly underrepresented (hyperrugose: *P*=0.0011, filiform: *P*=0.0016). At transfer 20, however, hyperrugose colonies were significantly underrepresented, while both circumscribed and filiform morphotypes were strongly overrepresented (*P*<0.0001). Transfer 30 showed no significant over- or underrepresentation of any individual morphotype from the expected count.

**Fig. 5.**
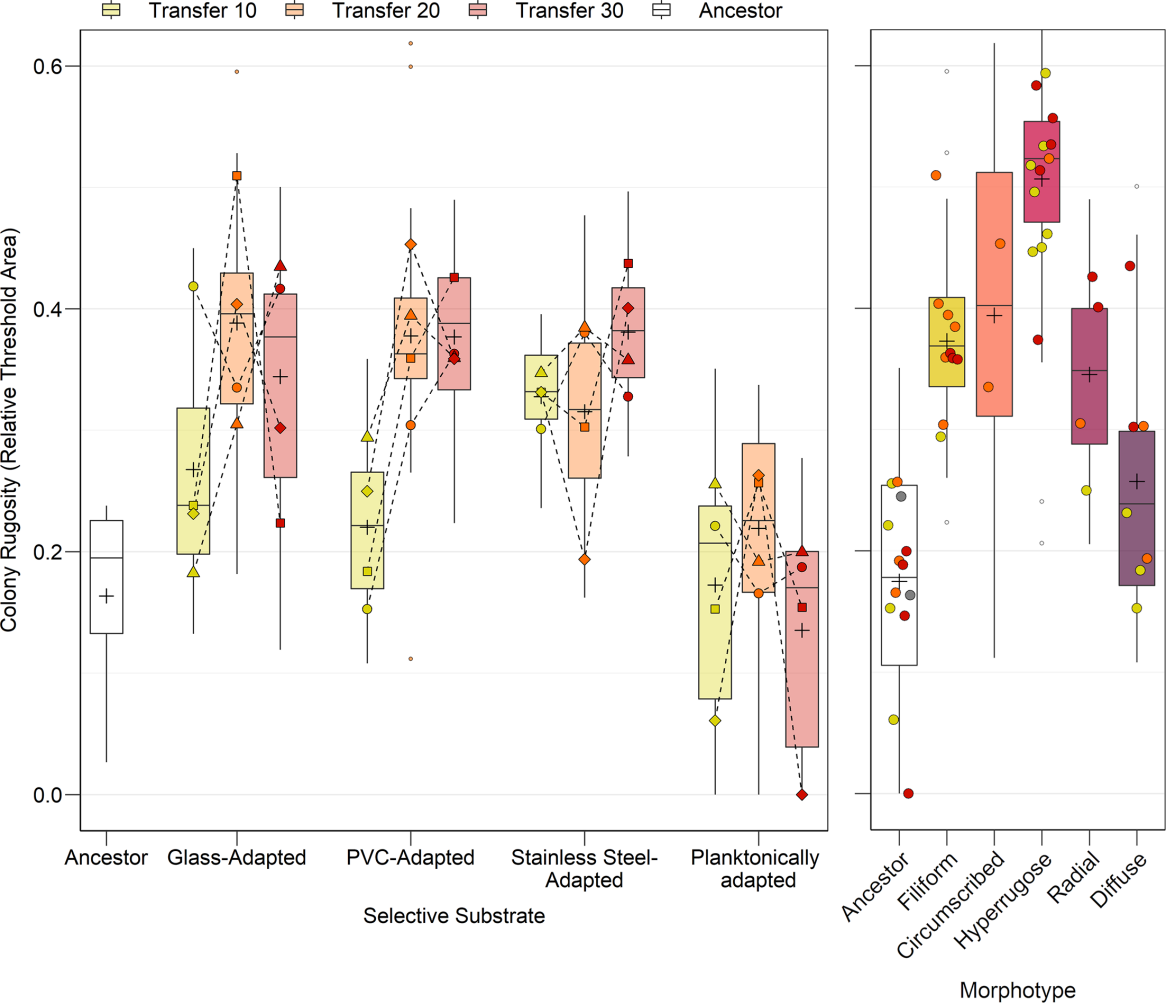
Colony rugosity morphometry of experimentally evolved lineages. The colony rugosity of each lineage was determined by measuring the coverage of rugose folds using a linear threshold model in Fiji and plotting the threshold area relative to the total colony area. Statistical differences in rugosity between selective substrates were determined by a two-way ANOVA. A significant main effect of selective substrate on rugosity was detected [*F*(4, 183)=47.622, *P*<0.0001], and a significant interaction effect was identified between substrate and timepoint [*F*(6, 183)=8.761, *P*<0.0001]; however, a main effect of timepoint was not detected [*F*(2, 183)=1.330, *P*=0.267]. Post hoc testing with Dunnett’s test identified that all biofilm substrates gave rise to higher rugosity than the ancestor (glass: *P*=0.0003, PVC: *P*=0.0196, stainless steel: *P*<0.0001), but not the planktonically adapted lineages (*P*=0.9925). According to Šidák’s post hoc test, the glass- and PVC-adapted lineages demonstrated a significant increase in rugosity between transfers 10 and 20 (PVC: *P*=0.0018, stainless steel: *P*=0.0016), with no further significant changes thereafter (glass: *P*=0.1357, PVC: *P*>0.9999). There was no increase in rugosity over time in the stainless steel-adapted lineages (*P*>0.9999). Colony rugosity also varied significantly by morphotype according to a one-way ANOVA [*F*(5, 179)=79.701, *P*<0.0001]. Post hoc testing with Tukey’s HSD test identified that all adapted morphotypes possessed significantly greater rugosity than the ancestor (circumscribed: *P*<0.0001, diffuse: *P*=0.0040, filiform: *P*<0.0001, hyperrugose: *P*<0.0001, radial: *P*<0.0001). The hyperrugose lineages had significantly greater rugosity than all other morphotypes (circumscribed: *P*=0.0266, filiform: *P*<0.0001, radial: *P*<0.0001, diffuse: *P*<0.0001), and the diffuse lineages were the least rugose among all morphotypes (circumscribed: *P*=0.0059, filiform: *P*<0.0001, hyperrugose: *P*<0.0001 radial: *P*=0.0420). Data are shown as mean coverage of rugosity relative to colony area, box limits show first and third quartiles, whiskers show ±1.5× interquartile range, large points show lineage mean and small points show outliers, *n*=16.

The colony rugosity of the experimentally evolved lineages was quantified by applying a global linear intensity threshold to colony images in Fiji (Fig. 5). The rugosity of the colony depended significantly on the morphotype observed (*P*<0.0001). Rugosity across the ancestral morphotype occupied ~15% of the colony area. All adapted morphotypes possessed significantly greater rugosity than the ancestor. The hyperrugose lineages had significantly greater rugosity than all other morphotypes, with ~50% coverage, and the diffuse lineages were the least rugose among all morphotypes at ~25% coverage. There was otherwise no significant difference in rugosity detected between the circumscribed, filiform and radial morphotypes, occupying between 35 and 40% of the area of the plate.

The colony rugosity also depended significantly on selective substrate (*P*<0.0001), but did not significantly change over the transfer series (*P*=0.267). Despite this, a significant interaction was identified between colony rugosity and substrate across the timepoint (*P*<0.0001). All biofilm substrates gave rise to higher rugosity than the ancestor, except for the planktonically adapted lineages. There was no increase in rugosity over time in the stainless steel-adapted lineages (*P*>0.9999); however, the PVC- and stainless steel-adapted lineages demonstrated a significant increase in rugosity between transfers 10 and 20 (PVC: *P*=0.0018, stainless steel: *P*=0.0016), with no further significant changes thereafter (PVC: *P*>0.9999, stainless steel: *P*=0.1357).

The ancestral strain formed a halo of weak bacterial growth surrounding the colony which occupied ~65% of the area of the agar plate (Fig. S3). The evolved biofilms, in addition to becoming more rugose, also demonstrated a significant reduction in their capacity to invade agar over time (*P*<0.0001). Furthermore, there was also a significant effect associated with selective substrate and agar invasion (*P*<0.0001) and a significant interaction effect between selective substrate and transfer timepoint with agar invasion (*P*<0.0001). The planktonically adapted lineages did not demonstrate a significant change in agar invasion relative to the ancestor at any timepoint (*P*>0.9999). At transfer 10, no significant reduction in agar invasion in the biofilm-adapted substrate was observed (*P*>0.9999); however, by transfer 20, the glass- and stainless steel-adapted lineages demonstrated significantly reduced agar invasion, but not in the PVC-adapted lineages (glass: *P*=0.0001, PVC: *P*>0.7520, stainless steel: *P*=0.0082), which only demonstrated reduced agar invasion at transfer 30 (*P*=0.0013). Once a significant reduction in agar invasion was observed, no further reductions were identified in subsequent timepoints (glass: *P*>0.9999, steel: *P*=0. 9275), and all conditions which selected for compromised invasion were comparable, invading ~20–35% of the agar surface. A significant effect of morphotype on agar invasion was also detected [*F*(5, 190)=20.29, *P*<0.0001]. All evolved morphotypes were significantly less invasive than the ancestor (circumscribed: *P*<0.0001, diffuse: *P*<0.0001, filiform: *P*<0.0001, hyperrugose: *P*<0.0001, radial: *P*=0.0013). The diffuse morphotype demonstrated significantly higher agar invasion than the filiform or the hyperrugose morphotype (filiform: *P*=0.0359, hyperrugose: *P*=0.0367); however, no other significant differences in agar invasion were detected between morphotypes.

### The cyclic-di-GMP signalling network is the main nidus of selection in experimentally evolved biofilm hyperproducers

A total of 156 unique SNPs were identified across all evolved populations. Of these, 108 (69.2%) were nonsynonymous, 16 (10.2%) were synonymous and 32 (20.5%) were located in noncoding regions. The average number of nonsynonymous polymorphisms per population was 1.7±1.3 (mean±sd). The mean number of mutations increased with subsequent timepoints from 1.3±1.2 at transfer 10, to 1.7±1.3 at transfer 20 to 2.4±1.2 at transfer 30. There was little difference in the mean number of mutations between selective substrates, with 2.0±1.5 in glass-adapted lineages, 2.1±1.4 in PVC-adapted lineages and 1.7±1.4 in stainless steel-adapted lineages. However, the planktonically adapted lineages possessed fewer SNPs with 1.3±1.2 mutations per population on average.

**Fig. 6.**
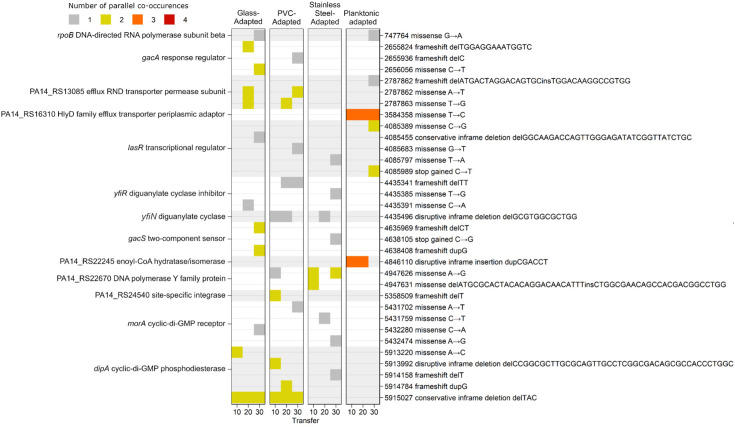
Gene targets under parallel selection in experimentally evolved lineages identified from Illumina short-read whole-genome sequencing data using snippy v4.6.0. Data are shown as the number of times a mutation co-occurred in the four parallel lineages sequenced, *n*=4.

Mutations in genes and pathways conserved across multiple parallel lineages were identified in order to explore the mechanisms of biofilm hyperproduction and phenotypic diversification observed in the evolved lineages. Very strong evolutionary parallelism towards selection for mutations in regulators of cyclic diguanylate (c-di-GMP) signalling or in genes transcriptionally regulated by c-di-GMP was identified across all the biofilm-adapted lineages independent of selective substrate (Fig. 6). This included mutations in the Gac/Rsm cascade response regulator *gacA* which demonstrated a Leu108 frameshift, Phe147 frameshift and Arg185Cys substitution, and its sensor kinase *gacS* which demonstrated a Leu56 frameshift, Val870 frameshift and early stop at Tyr767. *gacAS* mutations were observed in all glass-adapted lineages, however identified only in a single lineage adapted to PVC and stainless steel.

Mutations in the diguanylate cyclase inhibitor *yfiR* and diguanylate cyclase *yfiN* (originally named *tpbB* by Ueda and Wood [36])*,* both of which are components of the *yfiBNR* signalling complex, were observed in a single glass-adapted lineage and in two lineages adapted to PVC and stainless steel. This included a Pro178Gln substitution, Val176Gly substitution and Phe162 frameshift in *yfiR*, and a 12 bp in-frame deletion in *yfiN* which resulted in a Gly24 to Leu27 deletion in both lineages.

The bifunctional phosphodiesterase-diguanylate cyclase *morA* demonstrated several mutations in a single lineage of glass-, PVC- and stainless steel-adapted lineages including an Asn1148Lys substitution, Ser956Cys substitution and a His975Tyr substitution, respectively, which was later replaced by a Glu1213Gly substitution.

Mutations in the primary regulator of the motile-sessile switch in *P. aeruginosa*, the phosphodiesterase *dipA*, were identified in two glass-adapted lineages, including an in-frame Tyr875 deletion and a Thr272Pro substitution. Furthermore, the same Tyr875 deletion was observed in a PVC-adapted lineage alongside an Arg532 to Ala545 deletion in addition to a guanine duplication which resulted in an Ala794 frameshift. A Leu585 frameshift was also observed in a single steel-adapted lineage.

Mutations which did not demonstrate genotypic parallelism but nevertheless possessed hypothesized roles in the adaptive process based on broader phenotypic functions were also identified (Table S2). Mutations in chemoreceptor *wspA,* two-component sensor *wspE* and glutamate methylesterase *wspF* in the Wsp signal transduction pathway, involved in the motile-sessile switch, were identified in a glass- and PVC-adapted lineage including a 2 bp deletion resulting in a frameshift, and a 42 bp in-frame deletion and early stop, respectively. Similarly, in two putative c-di-GMP regulators, the EAL domain-containing PA14_RS18640 and GGDEF domain-containing PA14_RS22965, an early stop and a missense mutation were identified in one glass- and steel-adapted lineage, respectively. Finally, in one glass- and PVC-adapted lineage, mutations were identified in global regulators of flagellar motility including a missense mutation in *fleQ* and a frameshift mutation in *cheB*, respectively.

Several mutations were also identified in both planktonic- and biofilm-adapted lineages which likely reflect adaptation to culture conditions. This included mutations in the transcriptional regulator of the *las* quorum sensing system *lasR* in two planktonically adapted lineages and one glass-, PVC- and stainless steel-adapted lineage. Furthermore, in the resistance-nodulation-division efflux pump permease subunit PA14_RS13085, an AT to TG substitution was identified in two lineages of both glass- and PVC-adapted lineages, resulting in two missense mutations, whereas in a planktonically adapted lineage, a 16 bp indel resulted in a frameshift mutation at the same locus. Finally, a mutation in *rpoB* was identified in a single planktonic- and glass-adapted lineage. Mutations found exclusively in the planktonically adapted lineages included an Ile262Thr substitution in the PA14_RS16310 HlyD family efflux transporter periplasmic adaptor subunit in three planktonic lineages. Similarly, three planktonic lineages also possessed a 6 bp in-frame insertion in enoyl-coenzyme A hydratase/isomerase family protein PA14_RS22245, resulting in an Asp-Leu insertion between Leu186 and Ala187.

## Discussion

In this work, biofilms of *P. aeruginosa* PA14 were experimentally adapted in a selective environment which required repeated colonization of and dissemination from bead substrata. It was observed that this passage regime readily selected for biofilm hyperproduction in a stepwise manner. Within ten transfers, experimentally evolved mutants formed ~1.5× to 2× more biofilm than the ancestral strain, which increased to 2× to 3× more biofilm by transfer 30 irrespective of the substrate used for selection. Biofilm hyperproduction was therefore an effective mechanism to achieve fitness gains in an environment where a surface-associated lifestyle is under selection. Higher fitness in this environment was proportional to the degree of biofilm formation, likely by increasing the mutant’s ability to adhere, colonize and mechanically disseminate to a new substrate. Furthermore, acquisition of biofilm hyperproduction was not associated with compromised planktonic growth relative to planktonic lineages based on doubling times or viability in broth.

Biofilm hyperproduction was also not associated with altered susceptibility to a panel of clinically relevant antibiotics based on the MIC, which would identify constitutive lifestyle-independent changes in susceptibility, or MBEC, which accounts for biofilm-mediated antimicrobial tolerance [37]. Therefore, antimicrobial tolerance or intrinsic resistance was not inherently associated with adaptation to a biofilm lifestyle in this model system. Failure to achieve significant changes in intrinsic resistance in experimentally evolved biofilm hyperproducers has also been observed in *Salmonella* sv. Typhimurium [38]. In both this work and Trampari *et al*. [38], biofilms were grown in monoculture, and biomass production was used as the main metric of identifying biofilm hyperproducers. It is now recognized that the action of the extracellular matrix as a diffusion barrier which excludes antimicrobial compounds is not a dominant mechanism of biofilm-specific antimicrobial tolerance for many agents [39]. Instead, tolerance is likely a phenomenon related to the stratification of metabolic activity resulting in quiescence and the polymicrobial nature of real-world biofilms, with multiple taxa with disparate susceptibilities co-existing within the community.

**Fig. 7.**
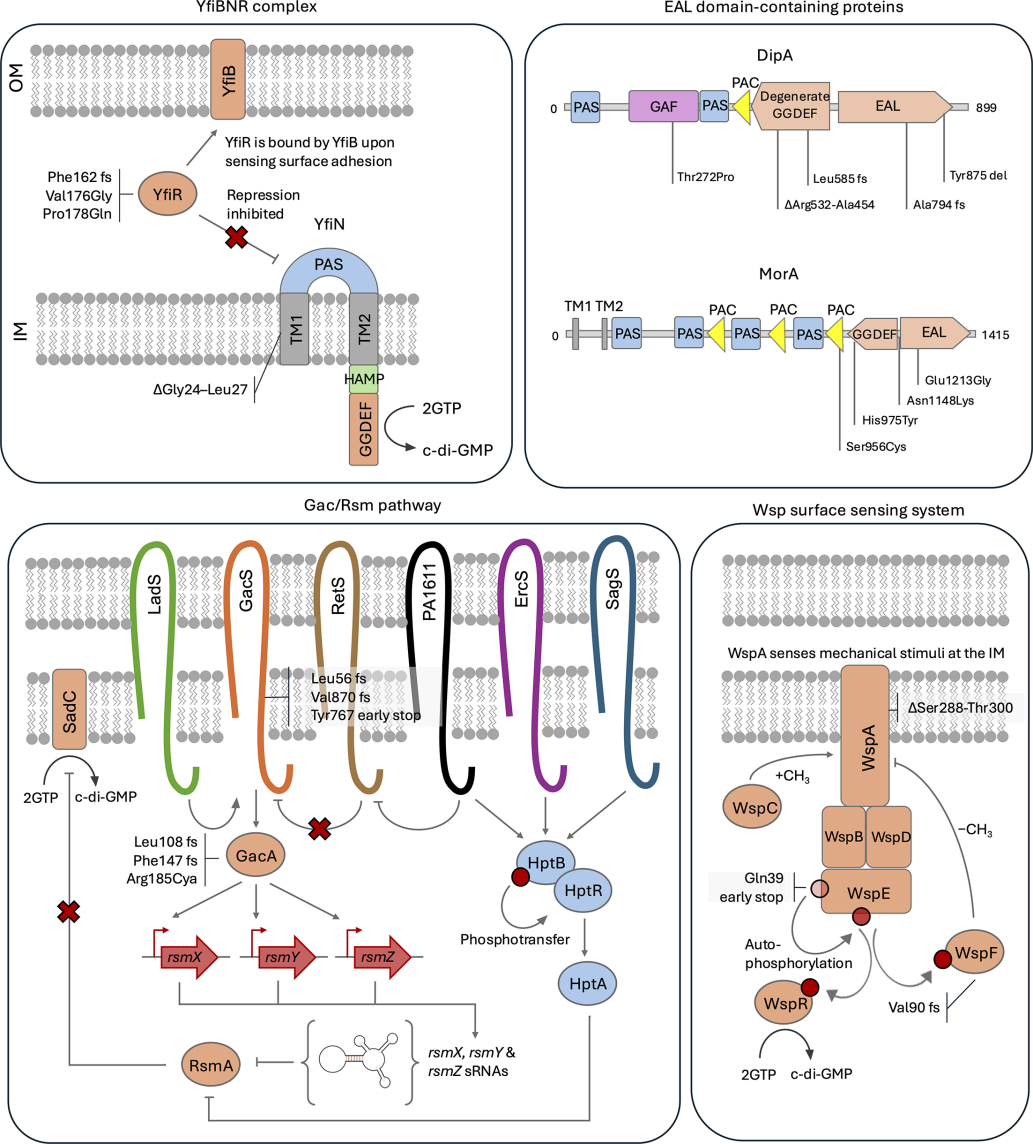
Pathways with mutations involved in c-di-GMP signalling identified in biofilm-adapted lineages. The YfiBNR system is a tripartite regulatory complex that modulates intracellular c-di-GMP levels in response to envelope stress. During surface adhesion, YfiB sequesters the periplasmic repressor *YfiR*, releasing the diguanylate cyclase YfiN to produce c-di-GMP. In this study, mutations in *yfiR* likely disrupted its ability to bind YfiN, while a Gly24–Leu27 in-frame deletion in the first transmembrane domain of *yfiN* may enhance its activity or stability, promoting biofilm hyperproduction. Mutations across multiple domains were also selected in MorA, a bifunctional diguanylate cyclase–phosphodiesterase, likely shifting its activity towards net c-di-GMP synthesis, and in DipA, a phosphodiesterase critical for biofilm dispersal, which likely results in the loss of function, preventing a return to a planktonic lifestyle. The Gac/Rsm pathway regulates the motile–sessile switch via the sensor kinase GacS and response regulator GacA, which activates transcription of the small noncoding RNAs (sRNAs) *rsmX*, *rsmY* and *rsmZ*. These sRNAs sequester the post-transcriptional repressor RsmA, which in turn lifts translational repression of the diguanylate cyclase SadC, leading to increased c-di-GMP production and transition to a sessile lifestyle. Multiple independent mutations were detected in *gacA* and *gacS,* which likely act to constitutively activate GacA thereby de-repressing SadC. Finally, the Wsp pathway detects mechanical stimuli at the inner membrane with the chemoreceptor WspA which, in turn, activates the sensor kinase WspE. WspE phosphorylates the GGDEF domain-containing response regulator WspR, promoting production of c-di-GMP. Normally, this is controlled through a feedback system with the methylesterase WspF, which is phosphorylated to an active state to negatively regulate the activity of WspA. In this study, a frameshift mutation was detected in *wspA,* which likely disrupts signal detection or triggers constitutive activation; a 14-residue in-frame deletion in *wspE* which may alter kinase function or specificity; and an early stop codon in *wspF* which may abolish its negative regulatory function, resulting in constitutive Wsp pathway activation.

Selective parallelism in independent lineages is a strong indication that a mutation is selectively advantageous and not a result of the random accumulation of neutral mutations; therefore, the evolutionary trajectories to biofilm hyperproduction were inferred by identifying mutations selected in multiple parallel lineages. There was no evidence for selective parallelism in noncoding or synonymous SNPs among biofilm-adapted lineages, indicating that non-synonymous mutations in coding sequences were the primary determinant of adaptation to a biofilm lifestyle rather than noncoding variants or silent mutations. Mutations in genes within the c-di-GMP signalling network were detected extensively in biofilm-adapted lineages (Fig. 7). These included the phosphodiesterase *dipA*, the bifunctional diguanylate cyclase/phosphodiesterase *morA,* the diguanylate cyclase *yfiN* and the diguanylate cyclase inhibitor *yfiR*. No associations between these mutations and selective substrate or the timepoint at which they became fixed were detected, although this was not tested statistically due to the sample size limited by the number of lineages in each condition.

The genes *yfiN* and *yfiR* are encoded in an operon with the lipoprotein *yfiB* to express the YfiBNR tripartite complex which senses unknown periplasmic signals to modulate c-di-GMP levels [40]. During planktonic growth, YfiR represses YfiN to inhibit its diguanylate cyclase activity. However, during surface adhesion, the lipoprotein YfiB undergoes a conformational change which allows it to sequester YfiR, leading to YfiN activation. *yfiBNR* mutations have been detected in *P. aeruginosa* populations isolated from the cystic fibrosis lung which conferred a c-di-GMP-dependent SCV phenotype with increased biofilm formation [41]. In the experimentally evolved biofilm hyperproducers in this study, three lineages possessed substitutions in the putative YfiN binding site of YfiR hypothesized by Malone *et al*. [42]. Consequently, it is likely that these mutations impair the capacity for YfiR to repress YfiN, thus increasing c-di-GMP synthesis. Furthermore, two additional lineages possessed a 12 bp in-frame deletion at position 71 of *yfiN*. The YfiR binding site of YfiN is believed to be present within the periplasmic domain which spans residues 35 to 161; however, the *yfiN* deletion mutation occurred in the first transmembrane helix [43]. Whether a truncated transmembrane helix alters the conformation of the periplasmic domain of YfiN to release it from regulatory control is not known. Mutations in the transmembrane helix domains of YfiN which confer insensitivity to YfiR have been observed [42]. However, this is the first report of a deletion polymorphism in *yfiN* with a role in biofilm hyperproduction.

The EAL domain-containing phosphodiesterase *dipA* was the most highly represented selective target in the biofilm-adapted lineages. *dipA* mutations have a well-documented role in biofilm formation; deletion of *dipA* results in overexpression of *psl*, limits flagella rotation and reduces biofilm dispersal [44,46]. Roy *et al*. [47] conducted the most comprehensive analysis of the role of *dipA* to date and identified domain-dependent involvement in biofilm formation. In this study, most substitution sites were localized in the catalytic domain of DipA spanning residues 460 to 885. The remaining substitution was present in the GAF module which Roy *et al*. [47] observed did not affect phosphodiesterase activity but still modulated biofilm formation through an unknown mechanism.

Three lineages demonstrated mutations in the c-di-GMP receptor *morA* which possesses both an EAL and GGDEF domain. In *P. aeruginosa* PAO1, MorA possesses preferential diguanylate cyclase activity, and deletion of *morA* results in a compromised ability to form biofilms [48,49]. Despite this, mutations in *morA* were seen in three separate experimentally evolved biofilm hyperproducers, which may reflect strain variation if the *morA* allele possessed by *P. aeruginosa* PA14 favours phosphodiesterase activity. Despite this, two mutations were observed in the C-terminal region of the GGDEF domain. Therefore, a greater understanding is required to elucidate the selective advantage associated with *morA* mutations in these biofilm hyperproducers.

To allow extracellular signals to regulate the intracellular c-di-GMP reservoir, signalling cascades bridge the interface between environmental sensors and c-di-GMP regulators [50]. One of the most important properties of the c-di-GMP network is the capacity for localized activation of signalling pathways. The intracellular c-di-GMP reservoir is not uniformly distributed across the cell, instead localized concentrations of c-di-GMP dictate activation of receptors [51]. Furthermore, signalling cascades are often associated with endogenous diguanylate cyclases or phosphodiesterases to create feedback systems [52]. Consequently, they are also often a target of selection for lifestyle-specific adaptation such as hypervirulent and biofilm hyperproducing phenotypes [53,54].

The Gac/Rsm pathway is a major regulator of the motile-sessile switch in *P. aeruginosa* which utilizes the lifestyle-specific orphan sensors SagS, ErcS and PA1611 to activate a signalling cascade leading to de-repression of the diguanylate cyclase SadC [20]. Consequently, mutations which de-repress the Gac/Rsm cascade can lead to biofilm hyperproduction. In the experimentally evolved biofilm hyperproducers, two lineages of glass-adapted and one lineage of stainless steel-adapted biofilms possessed unique mutations in *gacA* and *gacS*. No mutations in the secondary regulators of the Gac/Rsm cascade such as HptB, LadS or RetS were found. This seems paradoxical as the expression of *gacSA* must be maintained in order to de-repress the Gac/Rsm cascade [55]. All lineages with mutations in *gacSA* also possessed pre-existing mutations in the c-di-GMP synthesis regulators *dipA* at some timepoint. It is possible that in these lineages, constitutive inactivation of Gac/Rsm occurred to ameliorate excessive c-di-GMP synthesis, resulting from other mutations. In this experiment, dissemination to new beads was a selective pressure as important as colonization of the substrate. DipA is the main regulator of biofilm dispersal in *P. aeruginosa*; therefore, it is hypothesized that reducing c-di-GMP concentrations via inactivation of GacSA in dispersal-compromised biofilm hyperproducers may support transmission through the experiment [47].

Another major c-di-GMP-sensing regulator of the biofilm lifestyle in *P. aeruginosa* is the Wsp surface sensing system. It is able to sense adhesion via the chemoreceptor WspA which detects mechanical pressure on membranes [52]. Subsequently, activation of WspA supports the autophosphorylation of the histidine kinase WspE which in turn phosphorylates the diguanylate cyclase WspR and methylesterase WspF [19]. SCVs of *P. aeruginosa* isolated from a porcine wound infection model have been observed to carry mutations in *wspA* and *wspF* which act to increase the expression of WspR to confer biofilm hyperproduction [56]. In the experimentally evolved biofilm lineages, mutations in *wspA and wspF* were observed in a lineage of glass- and PVC-adapted biofilms. In this study, a 14 aa deletion of residues 285 to 298 flanked by a direct repeat region was observed in *wspA*. This region has been shown to contain one of two methylation sites through which WspF interacts with WspA, the deletion of which results in constitutive activation of WspA de-repressing WspR [57]. An early stop codon at residue 44 was also observed in *wspE*. While mutations in *wspE* in biofilm hyperproducers have been previously observed, none were loss-of-function mutations given the essential role of WspE for activation of WspR [58]. Therefore, the selection of inactivation of WspE seems paradoxical. Hickman *et al*. [19] did not observe compromised biofilm formation associated with the deletion of *wspE*, in spite of its role, presumably as functional redundancy within the c-di-GMP signalling network can compensate for the loss of Wsp expression. It is possible that, like the mutations in GacSA, the strain with a truncated *wspE* gene may be compensating for reduced capacity for dissemination by virtue of mutations in *dipA*.

In addition to increased biomass production, adaptation to a biofilm lifestyle was also associated with the acquisition of complex colony morphologies universally characterized by increased rugosity and decreased invasion of agar. In this study, five distinct morphotypes were identified. Rugosity is given rise by c-di-GMP-dependent expression of exopolysaccharide production; however, the mechanisms which regulate self-organization into complex architectures are not well understood [59,60]. Perhaps best characterized is the ‘wrinkly spreader’ phenotype of *Pseudomonas fluorescens* which repeatedly evolved after adaptation to a pellicle lifestyle at an air-liquid interface [61]. These mutants overproduce a cellulose-like polymer driven by constitutive activation of diguanylate cyclase WspR, to form rugose mats that facilitate oxygen availability in an oxygen-limited environment [62]. Importantly, this was not the only driver of the ruffled spreader morphotype, which was able to be recovered from a large array of mutations, all achieving comparable fitness [63]. This reveals a remarkably plastic selective landscape intrinsic to spatially structured or heterogeneous environments such as biofilms, in which multiple regulatory mutations accessing varied c-di-GMP pathways can generate convergent adaptive phenotypes.

In this work, biofilm hyperproduction was not associated with commitment to a specific morphology; instead, lineages underwent diversification and dynamically switched between morphotypes throughout the experiment. This defined repertoire of morphotypes evolved across multiple parallel lineages, and the individual morphotypes were not associated with specific mutations. Indeed, the observed colony morphologies may be higher-order phenotypic consequences associated with elevated intracellular c-di-GMP levels which orchestrate a complement of biofilm-associated behaviours which lead to the emergent morphological diversity described here. The capacity for rapid transition between phenotypic states has been demonstrated to be evolutionarily beneficial in *P. fluorescens* alternately selecting for biofilm-hyperproduction and biofilm-neutral phenotypes [64]. Lineages evolved a localized hypermutable locus with up to a 5-log increase in substitution rates through expansion of simple sequence repeats. Most notably, lineages able to perform this high-frequency switching between states were also more likely to acquire subsequent adaptive mutations. Therefore, this context-dependent phenotypic switching may imply that morphotypic volatility arises to enable populations to explore multiple adaptive configurations and rapidly switch between them as selection pressures shift.

In the evolved biofilms, the capacity to colonize the agar surface became progressively compromised as the experiment progressed in a morphotype-independent manner. Such invasion patterns are not characteristic of either swarming or swimming, instead appearing comparable to type IV pili-mediated twitching. However, the downregulation of type IV pili would seem counterproductive given their role in facilitating cell-matrix interactions [65,66]. Ribbe *et al*. [66] observed that the presence of Pel increased the strength of interaction between type IV pili and the surface. Consequently, the increased matrix production associated with biofilm hyperproduction may result in more type IV pili occupied with matrix binding, resulting in sterically hindered twitching motility. Therefore, impaired agar invasion appears as an emergent property not directly regulated by c-di-GMP, but as a biophysical consequence of matrix overexpression driven by high c-di-GMP production.

In conclusion, adaptation of *P. aeruginosa* to a biofilm lifestyle through experimental evolution was able to select for biofilm hyperproduction on bead substrata of three clinically and industrially relevant materials: glass, PVC and type-316 stainless steel. Biofilm hyperproduction was not associated with intrinsic resistance to antibiotics but did result in a significantly reduced susceptibility to high salinity and increased susceptibility to alkaline stress, although no specific mutations were found to be associated with either phenotype, instead appearing to be an intrinsic property given rise by biofilm hyperproduction itself. Mutations in the c-di-GMP signalling network were the main mechanism by which successive increases in biofilm formation were achieved, specifically the *dipA* phosphodiesterase, *yfiBNR* signalling complex, *morA* c-di-GMP receptor and the Gac/Rsm and Wsp signal transduction cascades. Furthermore, self-generated diversity was fundamental to the biofilm lifestyle, with dynamic switching between morphotypes being commonplace, despite conserved mechanisms of biofilm hyperproduction. The interface between fixed mutations and the heterogeneity elicited by adaptation to a biofilm lifestyle was not obvious, which may implicate a higher-order association, such as an epigenetic component, in this phenomenon. This work demonstrates that selection for biofilm hyperproduction gave rise to a conserved but volatile repertoire of morphotypes largely independent of the selective target. This provides important insights into the evolutionary dynamics within biofilms and identifies the need for further work to integrate understanding of fixed mutations and epigenetic components to understand the biology of biofilm adaptation.

## Supplementary material

10.1099/mic.0.001605 Uncited Supplementary Material 1.

## Abbreviations

AUC: area under the curve
MBEC: minimum biofilm eradication concentration
MIC: minimum inhibitory concentration
PVC: polyvinyl chloride
SCV: small colony variant
