# Supplementary material for: Cyclic-di-GMP signalling mutants drive ecological succession and self-generated diversity in experimentally evolved biofilms of Pseudomonas aeruginosa
Source: Microbiology (Reading). 2025 Sep 15;171(9):001605. doi: 10.1099/mic.0.001605 (PMC12440571; doi:10.1099/mic.0.001605)
Supplement: Uncited Supplementary Material 1. [file mic-171-01605-s001.pdf]

Table S1. Drug susceptibility of experimentally evolved endpoint populations of *P. aeruginosa* adapted to growth planktonically or as a biofilm on glass, PVC or stainless-steel substrates.

Selective Substrate		Minimum Inhibitory Concentration (μg/ mL)							Minimum Biofilm Eradication Concentration (μg/ mL)						
		Piperacillin	Ceftazidime	Meropenem	Aztreonam	Ciprofloxacin	Tobramycin	Colistin	Piperacillin	Ceftazidime	Meropenem	Aztreonam	Ciprofloxacin	Tobramycin	Colistin
Ancestor		2	2	0.25	2	0.06	2	0.5	128	64	16	32	1	32	32
Glass-adapted	Lineage 1	2	2	0.25	2	0.06	2	0.5	128	64	16	32	1	32	32
	Lineage 2	2	2	0.25	2	0.06	2	0.5	128	64	16	32	1	32	32
	Lineage 3	2	2	0.25	2	0.06	2	0.5	128	64	16	32	1	32	32
	Lineage 4	2	2	0.25	2	0.06	2	0.5	128	64	16	32	1	32	32
PVC-adapted	Lineage 1	2	2	0.25	2	0.06	2	0.5	128	64	16	32	1	32	32
	Lineage 2	2	2	0.25	2	0.06	2	0.5	128	64	16	32	1	32	32
	Lineage 3	2	2	0.25	2	0.06	2	0.5	128	64	16	32	1	32	32
	Lineage 4	2	2	0.25	2	0.06	2	0.5	128	64	16	32	1	32	32
Stainless Steel-adapted	Lineage 1	2	2	0.25	2	0.06	2	0.5	128	64	16	32	1	32	32
	Lineage 2	2	2	0.25	2	0.06	2	0.5	128	64	16	32	1	32	32
	Lineage 3	2	2	0.25	2	0.06	2	0.5	128	64	16	32	1	32	32
	Lineage 4	2	2	0.25	2	0.06	2	0.5	128	64	16	32	1	32	32
Planktonically-adapted	Lineage 1	2	2	0.25	2	0.06	2	0.5	128	64	16	32	1	32	32
	Lineage 2	2	2	0.25	2	0.06	2	0.5	128	64	16	32	1	32	32
	Lineage 3	2	2	0.25	2	0.06	2	0.5	128	64	16	32	1	32	32
	Lineage 4	2	2	0.25	2	0.06	2	0.5	128	64	16	32	1	32	32

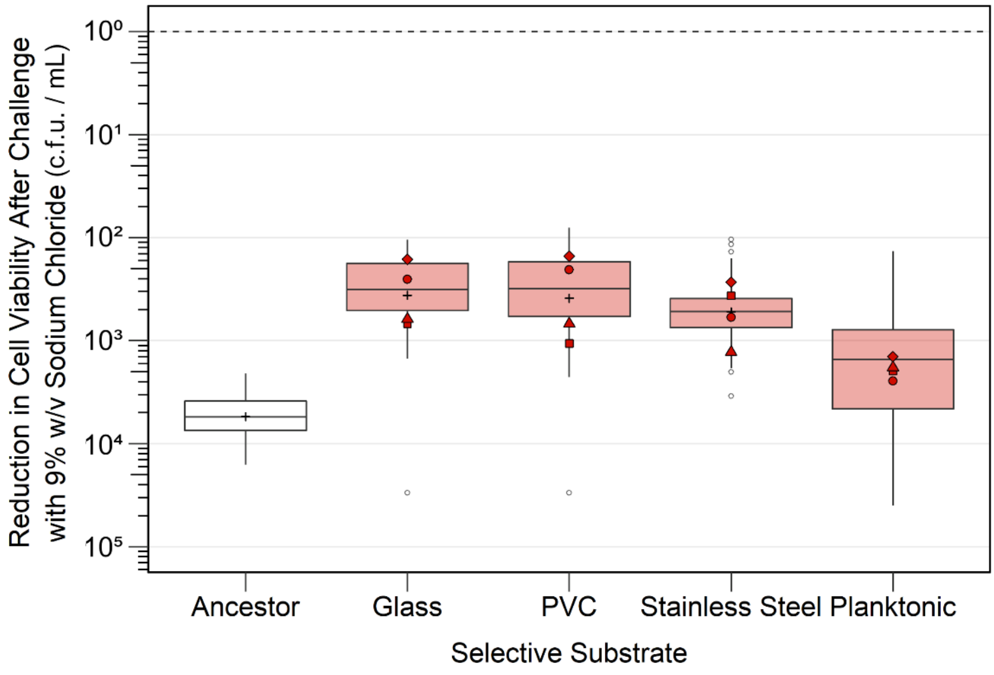

Figure S1. Tolerance of endpoint experimentally evolved lineages of *P. aeruginosa* adapted to growth planktonically or as a biofilm on glass, PVC or stainless-steel substrates to 9% w/v salt stress. Statistical differences between groups on viability at 9% w/v salt were determined via a one-way ANOVA. A significant main effect of selective substrate was detected ($F(4, 131) = 19.65$, $p < 0.0001$). Post-hoc testing with Tukey's HSD test identified that by transfer 30, all biofilm-adapted lineages were significantly more viable under salt stress than the ancestor (glass: $p < 0.0001$, PVC: $p < 0.0001$, stainless steel: $p < 0.0001$), but not the planktonically-adapted lineages ($p = 0.1020$). Data shown as reduction in viability in c.f.u./ mL, box limits show first and third quartiles, whiskers show ± 1.5× interquartile range, large free points show lineage mean, small free points show outliers, n = 4 per lineage.

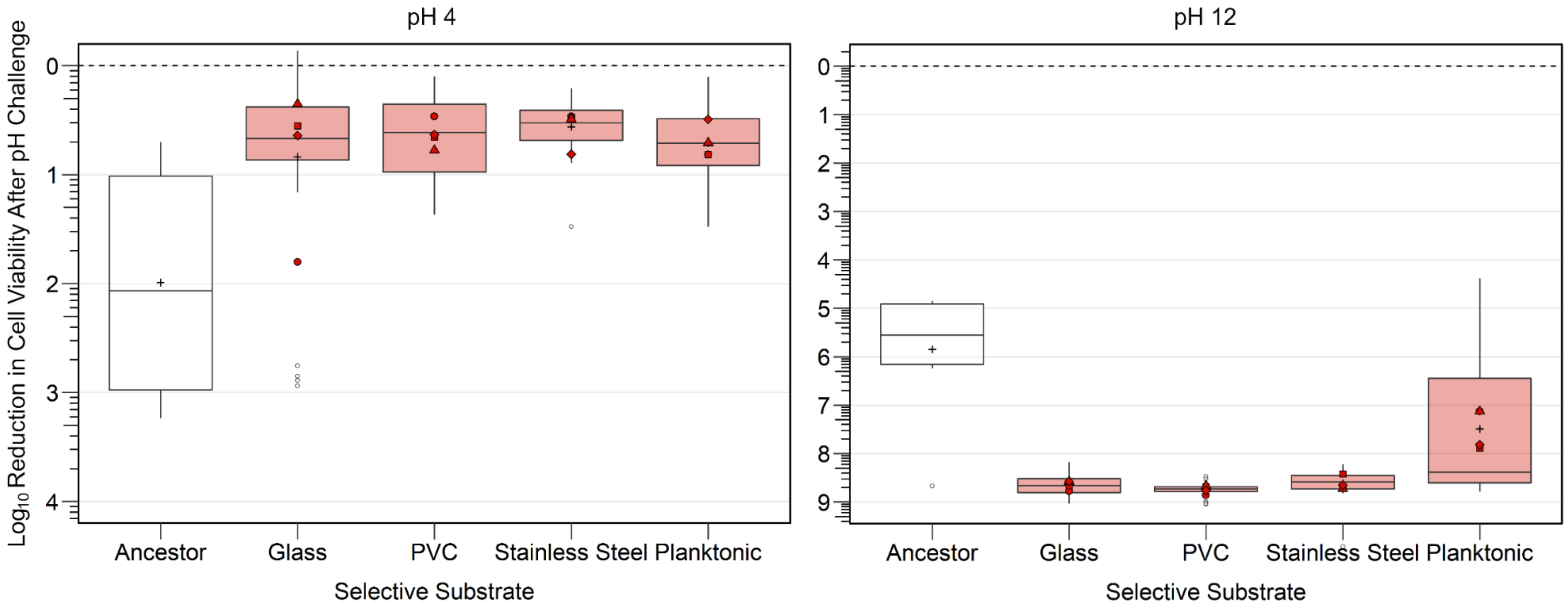

Figure S2. Tolerance to acid and alkaline stress of endpoint experimentally evolved lineages of *P. aeruginosa* adapted to growth planktonically or as a biofilm on glass, PVC or stainless-steel substrates. Statistical differences between groups on viability at pH 4 and 12 were determined via a two-way ANOVA. A significant difference between selective substrates was detected ($F(4, 262) = 12.72$, $p < 0.0001$) and a significant interaction between pH and selective substrate was also detected ($F(4, 262) = 42.21$, $p < 0.0001$). Dunnett's multiple comparisons test identified that all selective conditions at pH 4 demonstrated significantly higher viability than the ancestor according to post-hoc test (glass: $p < 0.0001$, PVC: $p < 0.0001$, stainless steel: $p < 0.0001$, planktonic: $p < 0.0001$) and at pH 12, all selective conditions demonstrated significantly lower viability than the ancestor (glass: $p < 0.0001$, PVC: $p < 0.0001$, stainless steel: $p < 0.0001$, planktonic: $p < 0.0001$). However, all biofilm-adapted lineages were significantly more susceptible than the planktonically-adapted lineages at pH 12 ($p < 0.0001$) but not at pH 4 (glass: $p = 0.8127$, PVC: $p = 0.9456$, stainless steel: $p = 0.7509$). Data shown as $\log_{10}$ reduction in viability in c.f.u., box limits show first and third quartiles, whiskers show ± 1.5× interquartile range, large free points show lineage mean, small free points show outliers, n = 4 per lineage.

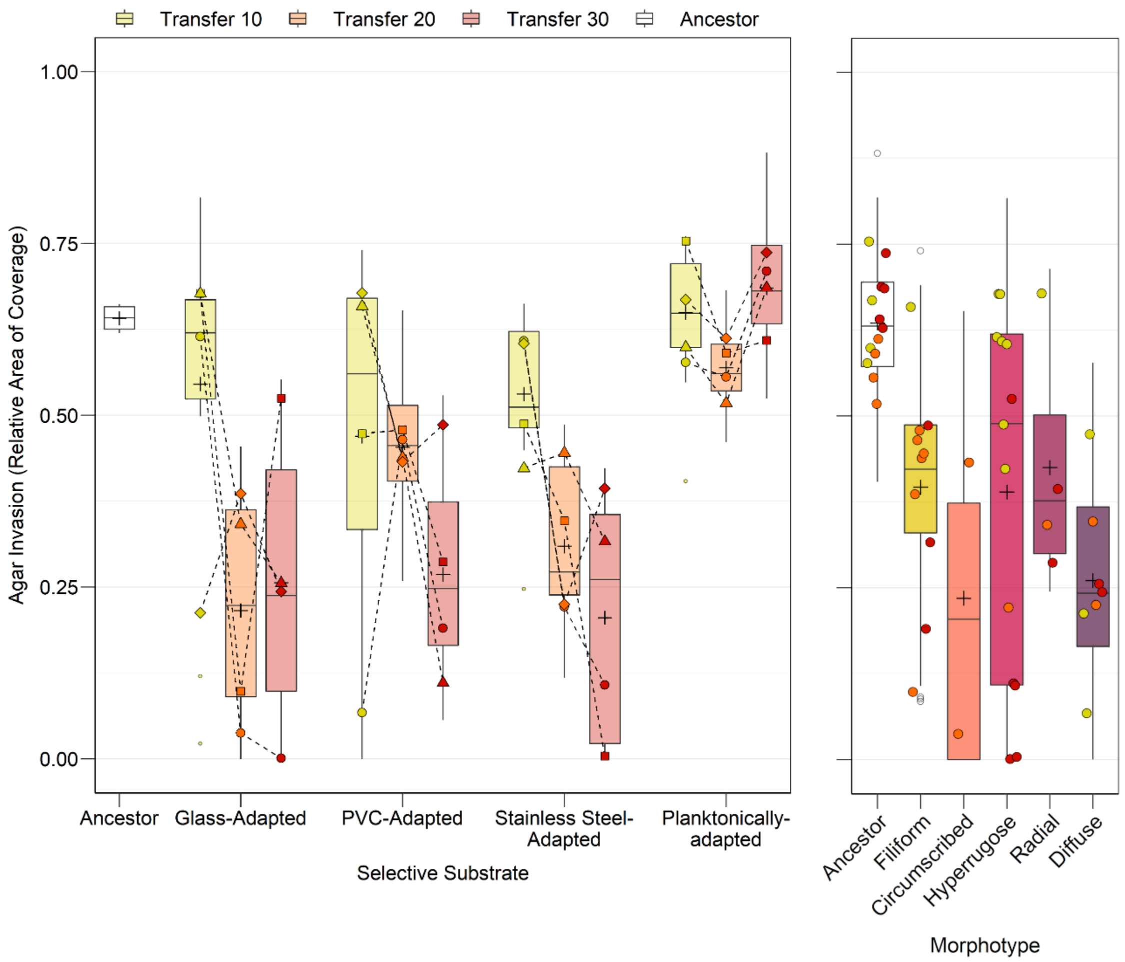

Figure S3. Agar invasion morphometry of experimentally evolved lineages of *P. aeruginosa* adapted to growth planktonically or as a biofilm on glass, PVC or stainless-steel substrates. The area of agar invasion was determined in Fiji by tracing the perimeter of the invasion halo, subtracting the colony area and plotting the invasion area relative to the total area of the agar plate. Statistical differences between selective substrates on agar invasion were determined by a two-way ANOVA. Significant main effects of number of transfers ($F(2, 183) = 28.568$, $p < 0.0001$) and selective substrate ($F(4, 183) = 30.281$, $p < 0.0001$) on extent of agar invasion were detected. Furthermore, a significant interaction effect between selective substrate and timepoint was also present ($F(6, 183) = 7.104$, $p < 0.0001$). According to Šidák's post-hoc test, at transfer 10, no significant reduction in agar invasion in the biofilm-adapted lineages was observed ($p > 0.9999$), however by transfer 20, a significant reduction in lineages adapted to glass and stainless steel was detected (glass: $p = 0.0001$, stainless steel: $p = 0.0082$). The PVC-adapted lineages demonstrated reduced agar invasion at exclusively at transfer 30 ($p = 0.0013$). A significant effect of morphotype on agar invasion was detected ($F(5, 190) = 20.29$, $p < 0.0001$). Tukey's HSD demonstrated that all evolved morphotypes were significantly less invasive than the ancestor ($p < 0.0001$). The diffuse morphotype demonstrated significantly higher agar invasion than either the filiform or the hyperrugose morphotype (filiform: $p = 0.0359$, hyperrugose: $p = 0.0367$), but there were otherwise no significant differences in agar invasion detected between morphotypes. Data shown as mean area of agar invasion relative to agar plate area, box limits show first and third quartiles, whiskers show ± 1.5× interquartile range, large free points show lineage mean, small free points show outliers, n = 4 per lineage.

Table S2. Non-synonymous substitutions in genes after substrate-specific adaptation which did not demonstrate genotypic parallelism but possess hypothesised roles in the adaptive process.

Lineage	Gene	Mutations	Transfer	Hypothesised Role
Glass-adapted				
Lineage 1	*wspF* Wsp signal transduction glutamate methylesterase	1412807 frameshift delTT	30	Motile-sessile switch
Lineage 3	PA14_RS18640 EAL domain-containing protein	4083502 stop gained C→T	20	c-di-GMP regulation
Lineage 4	*fleQ* transcriptional regulator	4460924 missense A→G	30	Flagellar motility
PVC-adapted				
Lineage 2	*wspA* Wsp signal transduction system chemoreceptor	1406486 conservative inframe deletion delTCCAAGCAGCAGCAGGCCACCGCCACCGAAACCGCCGCGACC	30	Motile-sessile switch
	wspE Wsp signal transduction system sensor histidine kinase	1409866 stop gained G→T	30	Motile-sessile switch
Stainless steel-adapted				
Lineage 2	*cheB* chemotaxis protein	476993 frameshift dupGA	30	Flagellar motility
Lineage 3	PA14_RS22965 GGDEF domain-containing protein	5024358 missense G→T	30	c-di-GMP regulation
